# Adverse Effect of Blue Light on DNA Integrity Is Accelerated by 5-Aminolevulinic Acid in HaCaT Human Keratinocyte Cells and B16F1 Murine Melanoma Cells

**DOI:** 10.3390/biology11121743

**Published:** 2022-11-30

**Authors:** Taiki Sato, Kazuomi Sato

**Affiliations:** 1 Graduate School of Agriculture, Tamagawa University, 6-1-1 Machida, Tokyo 194-8610, Japan; 2 Biosystems & Biofunctions Research Center, Tamagawa University Research Institute, 6-1-1 Machida, Tokyo 194-8610, Japan

**Keywords:** 5-ALA, blue light, DNA damage, comet assay, melanoma, keratinocyte

## Abstract

**Simple Summary:**

5-aminolevulinic acid (5-ALA) is a non-proteinogenic amino acid. Several reports show its beneficial effects, such as antiviral activity against COVID-19, anticancer effects, and a reduction in fatigue. In addition, 5-ALA acts as a precursor to a photosensitizer used for photodynamic therapy; however, 5-ALA may cause DNA damage in mammalian cells. In this study, we evaluated the effect of blue light and 5-ALA on DNA damage using mouse melanoma cells and human keratinocyte cell lines. We performed comet assays to assess DNA double-strand breaks (DSB) and single-strand breaks (SSB). Our results demonstrate that genomic DNA of both cancer cells and non-cancer cells was severely damaged by blue light irradiation in the presence of 5-ALA.

**Abstract:**

Several studies have suggested the potential benefits of 5-aminolevulinic acid (5-ALA)-based photodynamic therapy (PDT). 5-ALA is a precursor of heme, which generates reactive oxygen species (ROS) following photoirradiation. Some reports indicate that blue light induces intracellular ROS production. In the present study, we elucidated the effects of blue light and 5-ALA on DNA integrity in B16F1 murine melanoma and human keratinocyte HaCaT cells using a variety of comet assay techniques. Co-treatment with blue light and 5-ALA significantly decreased cell viability in both cell lines. A neutral comet assay was performed to assess DNA double-strand break (DSB) formation and blue light and 5-ALA caused DSBs. We also performed an alkali comet assay to detect single-strand breaks (SSB) and alkali labile sites (ALS). The results indicated that 5-ALA accelerated blue light-induced SSB formation. In addition, modified comet assays were done using two types of enzymes to evaluate oxidative DNA damages. The results indicated that blue light and 5-ALA generated oxidized purine and pyrimidines in both cell lines. In summary, co-treatment with 5-ALA and photoirradiation may cause unexpected DNA damage in cells and tissues.

## 1. Introduction

Numerous factors have been shown to aggravate DNA integrity. For example, mycotoxins, such as ochratoxin A, induces various types of DNA damage in animal tissues and cells [1]. Recently, Muthumalage et al. demonstrated that E-cigarette-flavored pods induce DNA damage in lung cells [2]. Ultraviolet (UV) radiation can induce DNA damage directly and indirectly [3]. Direct absorption of UV photons causes DNA damage including cyclobutene pyrimidine dimers (CPD) and 6-4 photoproducts (6-4PP) [4]. UV light also induces indirect DNA damage, such as 7,8-dihydro-8-oxoguanine (8-OxoG), which results in single-strand breaks (SSB) [5]. On the other hand, double-strand breaks (DSB) can occur in response to the repair of SSB, but are not caused by UV radiation directly [3].

5-aminolevulinic acid (5-ALA), which is a precursor of protoporphyrin IX (PPIX) in the heme biosynthesis pathway, has been reported to exert some positive biological effects [6]. Hijioka et al. reported that 5-ALA prevents neurodegeneration by suppressing oxidative stress in a rat model of Parkinson’s disease [7]. Additionally, 5-ALA enhances the activity of oxidative stress-related genes, such as nuclear factor erythroid 2-related factor 2 (Nrf2) and heme oxygenase-1 (HO-1), and protects bovine mammary epithelial cells from heat stress [8]. In cancer cells, PPIX tends to accumulate in the cells because of the low ferrochelatase activity, which converts PPIX into heme [9]. Thus, 5-ALA has been used for photodynamic therapy (PDT). PPIX enhances intracellular reactive oxygen species (ROS) levels following light exposure, which results in tumor cell death. Therefore, 5-ALA PDT is considered a novel cancer therapy. 

Previously, we found that blue and green light induce intracellular ROS accumulation and subsequent cell death of melanoma cells [10]. Several studies have also shown that 5-ALA PDT with blue light prevents cancer cell growth and is a promising option for anticancer therapy [11,12]; however, there is some concern with respect to the risks to genome integrity when exposing normal cells to 5-ALA PDT. Therefore, the aim of this study was to determine the effects of blue light and 5-ALA on DNA integrity in B16F1 murine melanoma cells and HaCaT normal human keratinocytes cells. We performed several variations of single cell gel electrophoresis (comet assay) to evaluate DNA damage. The neutral comet assay represents one technique to detect DSB in single cells [13,14], whereas the alkaline-modified comet assay can detect SSB and alkaline labile sites (ALS) [15]. Moreover, the enzyme-modified comet assay has enabled the detection of oxidative DNA damage using the bacterial repair enzymes formamidopyrimidine DNA glycosylase (Fpg) and endonuclease III (Endo III), which release damaged purines and pyrimidines, respectively [16]. However, the shortcoming of the methods is that they cannot distinguish between DSB and SSB in the same cells. Enciso et al. developed an innovative method, the two-tailed comet assay (TT-comet assay), which differentiates between DSB and SSB in sperm cells [17]. Here, we adopted the TT-comet assay to differentiate blue light and 5-ALA-induced DSB and SSB using B16F1 melanoma and normal HaCaT cells. 

## 2. Materials and Methods

### 2.1. Cell Culture

HaCaT cells (Cosmob Bio, Tokyo, Japan) and B16F1 melanoma cells (RIKEN BioResource Center, Tsukuba, Japan) were cultured in Dulbecco’s modified Eagle’s medium (Sigma, St. Louis, MO, USA) supplemented with 10% fetal bovine serum, 50 U/mL penicillin, and 100 µg/mL streptomycin at 37 °C in a humidified atmosphere containing 5% CO_2_. An LED apparatus was constructed in a CO_2_ incubator. In this study, we used blue LEDs (465 nm; A.S. Tech Corp., Osaka, Japan), and blue light treatments were performed at a light intensity of 50 W/m^2^. Information on the irradiance of the blue LED lamps is available from our previous study [10].

### 2.2. MTT Assay

To measure cell proliferation and viability, the 3-(4.5-dimethylthiazol-2-yl)-2,5-diphenyltetrazolium bromide (MTT) assay was performed. HaCaT cells and B16F1 melanoma cells were seeded into 35 mm dishes at a density of 2.0 × 10^4^ cells per dish. After a 24 h incubation, the cells were pretreated with 1 mM 5-aminolevulinic acid hydrochloride (5-ALA) (Sigma-Aldrich, St. Louis, MO, USA) for 1 h followed by blue light irradiation for 1 h. After irradiation, the cells were washed with PBS and the culture media was replaced. After incubation for 72 h, 50 µL of 5 mg/mL MTT (Sigma) was added and incubated for 3 h and 30 min at 37 °C. Finally, the precipitated formazan was dissolved with DMSO and the absorbance was measured at 590 nm using a microplate reader.

### 2.3. Neutral Comet Assay 

HaCaT or B16F1 cells were seeded into 60 mm dishes at a density of 2.5 × 10^5^ cells per dish. After a 24 h incubation, the cells were pretreated with 1 mM 5-ALA for 1 h followed by blue light irradiation for 1 h. After irradiation, both attached and detached cells were collected, washed with PBS, mixed with 1% low melting agarose in PBS, and mounted onto agarose-coated glass slides. The slides were immersed in lysis solution (2.5 M NaCl, 100 mM EDTA, 10 mM Tris, 10% dimethyl sulfoxide, and 0.1% Triton X-100) for 60 min at 4 °C, and then immersed in neutral electrophoresis buffer (25 mM Tris, 0.75 M sodium acetate, pH 9.0) for 30 min. After electrophoresis for 15 min at 45 V, the slides were immersed in DNA precipitation buffer for 30 min and then immersed in 70% ethanol for 30 min. The slides were stained with SYBR Gold (Thermo Fisher Scientific, Waltham, MA, USA). The comets were observed under an EVOS Cell Imaging System (Thermo Fisher Scientific). To assess DNA damage, the tail moment, %DNA in tail, and tail length of at least 100 cells on each slide were scored using OpenComet software v1.3.1 [18].

### 2.4. Alkaline Comet Assay

The alkaline comet assay can detect DSB, SSB, and ALS [19]. Following the same procedure as the neutral comet assay, the slides were immersed in lysis solution for 30 min at 4 °C. The slides were then immersed in alkaline unwinding solution (0.2 M NaOH, 1 mM EDTA) for 20 min in the dark at room temperature. Following electrophoresis, the comets were stained and evaluated as in the neutral comet assay section.

### 2.5. Two-Tailed Comet Assay (TT-Comet Assay)

To determine the effect of blue light and 5-ALA on DSB and SSB formation separately, we used the TT-comet assay as described by Palazzese et al. [20] with some modifications. The TT-comet assay is the only assay that discriminates SSB from DSB [21]. HaCaT or B16F1 melanoma cells were seeded into 35 mm dishes at a density of 5 × 10^4^ cells per dish. After incubation, the cells were pretreated with 1.0 mM 5-ALA for 1 h followed by blue light irradiation for 1 h. After treatment, the cells were lysed by the same method as that of the neutral comet assay. Electrophoresis was performed for 45 V, 15 min in neutral electrophoresis buffer, the slides were washed in ice-cold 0.9% NaCl briefly, and immersed into alkaline solution (30 mM NaOH, 1 M NaCl) for 2.5 min. A second electrophoresis was performed in alkaline electrophoresis buffer (30 mM NaOH, 1 mM EDTA) for 12 min at 20 V, with the slides oriented vertically as the first electrophoresis. After secondary electrophoresis, the slides were immersed into neutralization buffer (0.4 M Tris-HCl, pH 7.5) for 5 min. Then, the slides were dehydrated in increasing concentrations of ethanol (70% and 100% EtOH), air dried, and stained as described for the neutral comet assay. The type of DNA damage was classified as either DSBs (left-right migration) or SSBs (up-down migration). To evaluate the DNA damage in the TT-comet assay, we measured the whole comet length of the x-axis (for detect DSB) and y-axis (for detect SSB), respectively. 

### 2.6. Enzyme-Modified Comet Assay

Bacterial repair enzymes Fpg and Endo III were used in the enzyme-modified comet assay. The Fpg and Endo III-modified comet assay can detect oxidized purines and pyrimidines, respectively [22].

B16 melanoma and HaCaT cells were seeded into 35 mm dishes at a density of 5 × 10^4^ cells per dish. After a 24 h incubation, the cells were pretreated with 0.2 mM 5-ALA for 1 h followed by blue light irradiation for 1 h. After treatment, the cells on the slides were lysed as the same method as that of neutral comet assay. Then, slides were washed with enzyme reaction buffer (40 mM HEPES, 0.1 M KCl, 0.5 mM EDTA, 0.2 mg/mL BSA). Fpg (1:2000, New England Biolabs, Ispwich, MA, USA) and Endo III (1:1250, New England Biolabs) were diluted in enzyme reaction buffer and the slides were incubated in enzyme solution for 30 min at 37 °C in the dark. After enzyme treatment, the slides were immersed in alkaline unwinding solution for 20 min in the dark at room temperature. Following electrophoresis, the comets were stained and evaluated as in the neutral comet assay. To calculate the net Fpg- and Endo III-sensitive sites, we adopted a previously described method [23].

### 2.7. Statistical Analysis

Statistical significance was analyzed with a one-way ANOVA for MTT assay, and a non-parametric Kruskal–Wallis test for comet assay using GraphPad Prism 9 software. Two levels of statistical significance relative to the control (unirradiated) group were defined: ^#^ *p* < 0.05, ^##^ *p* < 0.01. * *p* < 0.05, ** *p* < 0.01 was considered significant.

## 3. Results

### 3.1. Effect of Blue Light and 5-ALA on Cell Viability

**Figure 1:**
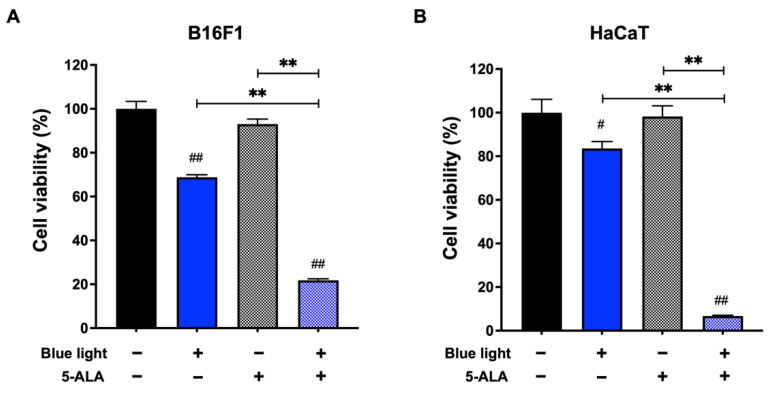
Effect of blue light and 5-ALA on the viability of B16F1 melanoma and HaCaT cells. The MTT assay was performed to assess the viability of (**A**) B16F1 melanoma and (**B**) HaCaT cells. Both cell lines were pretreated with 1 mM 5-ALA for 1 h followed by irradiation with 50 W/m^2^ blue light for 1 h in the presence of 5-ALA. After treatment, the culture media were replaced and incubated for 72 h until the MTT assay. Data are presented as the mean ± SEM obtained from at least four independent experiments. ^#^ *p* < 0.05, ^##^ *p* < 0.01 versus control, ^##^ *p* < 0.01, ** *p* < 0.01 was considered significant.

First, we performed an MTT assay to evaluate the effect of co-treatment with blue light and 5-ALA on the viability of B16F1 melanoma and HaCaT cells. As shown in Figure 1, 5-ALA showed no significant cell toxicity to either cell line. Compared with the unirradiated control, cell viability was reduced in the blue light exposed cells. Moreover, co-treatment with blue light and 1 mM 5-ALA resulted in a marked decrease in the viability of both cell lines.

### 3.2. Co-Treatment with Blue Light and 5-ALA Induces DNA Strand Breaks in B16F1 and HaCaT Cells 

**Figure 2:**
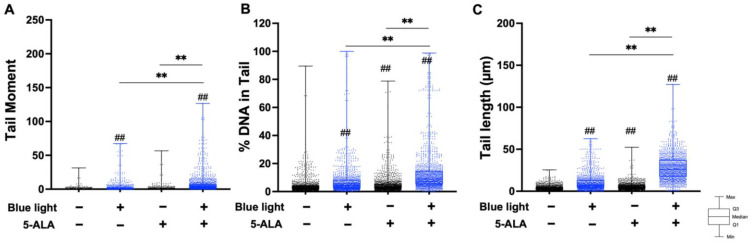
Effect of blue light and 5-ALA on DNA DSB formation in B16F1 melanoma cells. A neutral comet assay was performed to detect DSB formation. B16F1 melanoma cells were pretreated with 1 mM 5-ALA for 1 h followed by irradiation with 50 W/m^2^ blue light for 1 h in the presence of 5-ALA. (**A**) Tail moment, (**B**) %DNA in tail, and (**C**) tail length parameters were evaluated using OpenComet software. Data are presented as the medians obtained from at least four independent experiments. ^##^ *p* < 0.01 versus control, ^##^ *p* < 0.01, ** *p* < 0.01 was considered significant.

**Figure 3:**
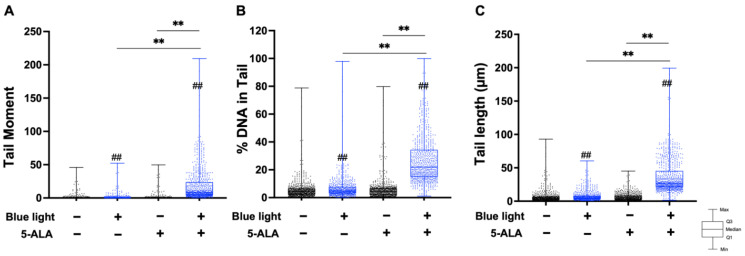
Effect of blue light and 5-ALA on DNA DSB formation in HaCaT cells. HaCaT cells were treated with blue light and 5-ALA and a neutral comet assay was performed. HaCaT cells were pretreated with 1 mM 5-ALA for 1 h followed by irradiation with 50 W/m^2^ blue light for 1 h in the presence of 5-ALA. (**A**) Tail moment, (**B**) %DNA in tail, and (**C**) tail length parameters were evaluated using OpenComet software. Data are presented as the medians obtained from at least four independent experiments. ^##^ *p* < 0.01 versus control, ^##^ *p* < 0.01, ** *p* < 0.01 was considered significant.

To evaluate the effect of blue light and 5-ALA on DNA integrity, we conducted a neutral comet assay to detect DSBs. As shown in Figure 2, co-treatment with blue light and 5-ALA significantly induced DNA DSBs formation in B16F1 melanoma cells. Moreover, blue light-only treated cells also exhibited DSB formation. Although the tail moment parameter did not change after 5-ALA-only treatment, tail length elongation was observed. In contrast, only co-treatment induced DNA DSBs formation in HaCaT cells (Figure 3). 

**Figure 4:**
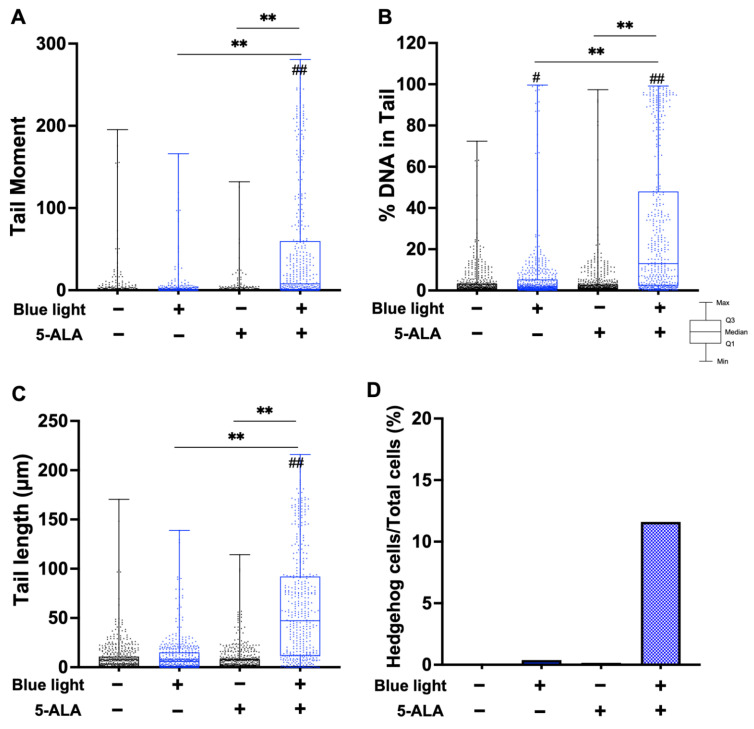
Effect of blue light and 5-ALA on DSBs, SSBs, and ALS formation in B16F1 melanoma cells. The alkaline comet assay was used to detect DSBs, SSBs, and ALS formation. B16F1 melanoma cells were pretreated with 1 mM 5-ALA for 1 h followed by irradiation with 50 W/m^2^ blue light for 1 h in the presence of 5-ALA. (**A**) Tail moment, (**B**) %DNA in tail, and (**C**) tail length parameters were evaluated using OpenComet software. (**D**) %Hedgehog cells were calculated from the number of hedgehog cells in the total cells including hedgehog cells. Data are presented as the medians obtained from at least four independent experiments. ^#^ *p* < 0.05, ^##^ *p* < 0.01 versus control, ^##^ *p* < 0.01, ** *p* < 0.01 was considered significant.

**Figure 5:**
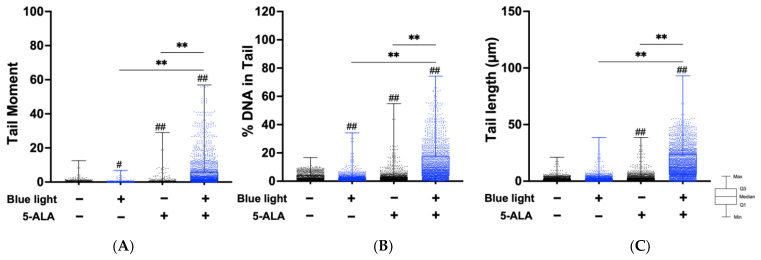
Effect of blue light and 5-ALA on DSBs, SSBs, and ALS formation in HaCaT cells. The alkaline comet assay was used to detect DSBs, SSBs, and ALS formation. HaCaT cells were pretreated with 1 mM 5-ALA for 1 h followed by irradiation with 50 W/m^2^ blue light for 1 h in the presence of 5-ALA. (**A**) Tail moment, (**B**) %DNA in tail, and (**C**) tail length parameters were evaluated using OpenComet software. Data are presented as the medians obtained from at least four independent experiments. ^#^ *p* < 0.05, ^##^ *p* < 0.01 versus control, ^##^ *p* < 0.01, ** *p* < 0.01 was considered significant.

Next, we performed an alkaline comet assay to assess SSB and ALS formation. As shown in Figure 4, the tail moment, tail DNA content, and tail length were significantly increased by co-treatment with blue light and 5-ALA in B16F1 melanoma cells. The same results were observed in a series of experiments using HaCaT cells (Figure 5). In both cell lines, blue light or 5-ALA treatment alone did not affect SSB formation. 

Cells may be classified into scorable cells and highly damaged nonscorable cells. These abnormal cells are referred to as ‘hedgehog’ or ‘cloud’ or ‘ghost’ cells [24,25]. We counted hedgehog cells in the total comets. As shown in Figure 4D, hedgehog comets accounted for over 10% of the total cells following blue light and 5-ALA co-treatment of B16F1 melanoma cells. In contrast, we did not detect hedgehog comets in HaCaT cells. 

### 3.3. TT-Comet Assay

**Figure 6:**
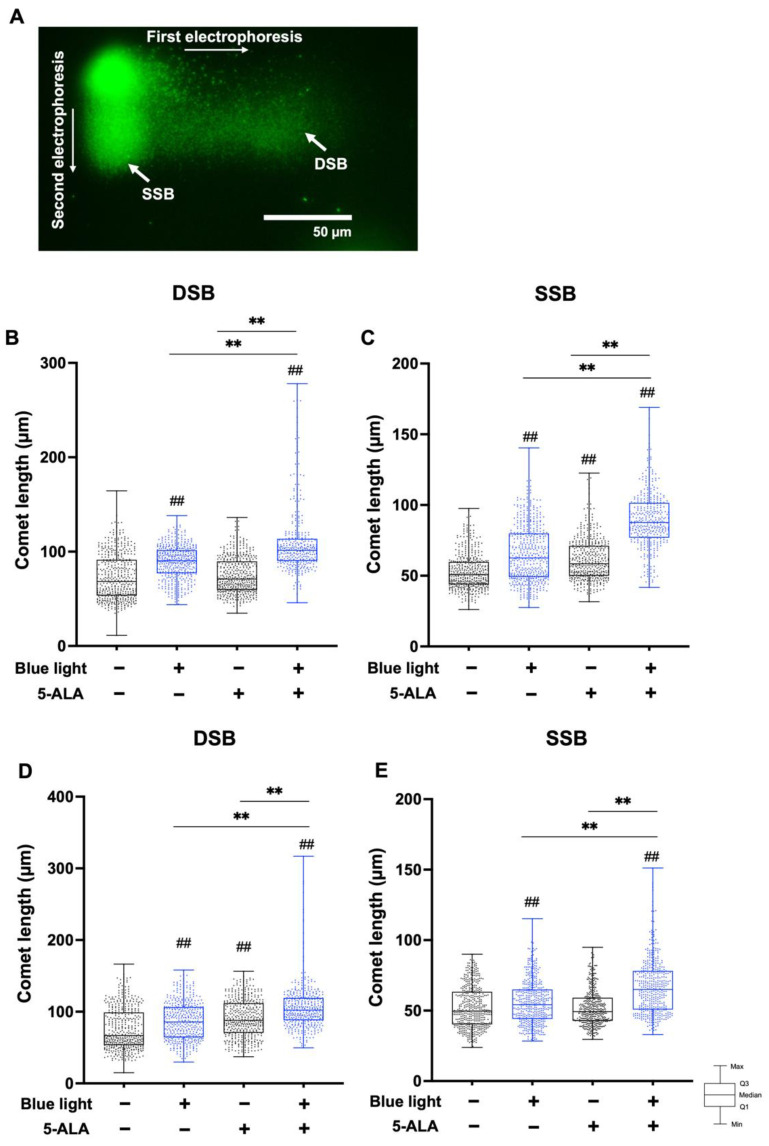
TT-comet assay for the detection of DSB and SSB formation in B16F1 melanoma and HaCaT cells. The TT-comet assay was used to detect DSBs and SSBs separately. B16F1 and HaCaT cells were pretreated with 1 mM 5-ALA for 1 hfollowed by irradiation with 50 W/m^2^ blue light for 1 h in the presence of 5-ALA. After treatment, electrophoresis was done under neutral conditions followed by electrophoresis under alkaline conditions. (**A**) Image of TT-comet assay showing B16F1 melanoma cells after 5-ALA and blue light co-treatment. (**B**) DSBs and (**C**) SSBs in B16F1 melanoma cells and (**D**) DSBs and (**E**) SSBs in HaCaT cells are indicated as comet length. Data are presented as the mean medians obtained from at least four independent experiments. ^##^ *p* < 0.01 versus control, ^##^ *p* < 0.01, ** *p* < 0.01 was considered significant.

To differentiate blue light- and 5-ALA-induced DSBs and SSBs, we performed the TT-comet assay. The TT-comet assay enables the detection of DSB and SSB formation separately from the migration length to the x-axis and y-axis, respectively (Figure 6A). In B16F1 melanoma cells, both DSB and SSB formation were enhanced by co-treatment with blue light and 5-ALA (Figure 6B,C). As shown in Figure 6D,E, DSB and SSB were also enhanced by blue light and 5-ALA co-treatment in HaCaT cells. 

### 3.4. Fpg- and Endo III-Modified Comet Assay

**Figure 7:**
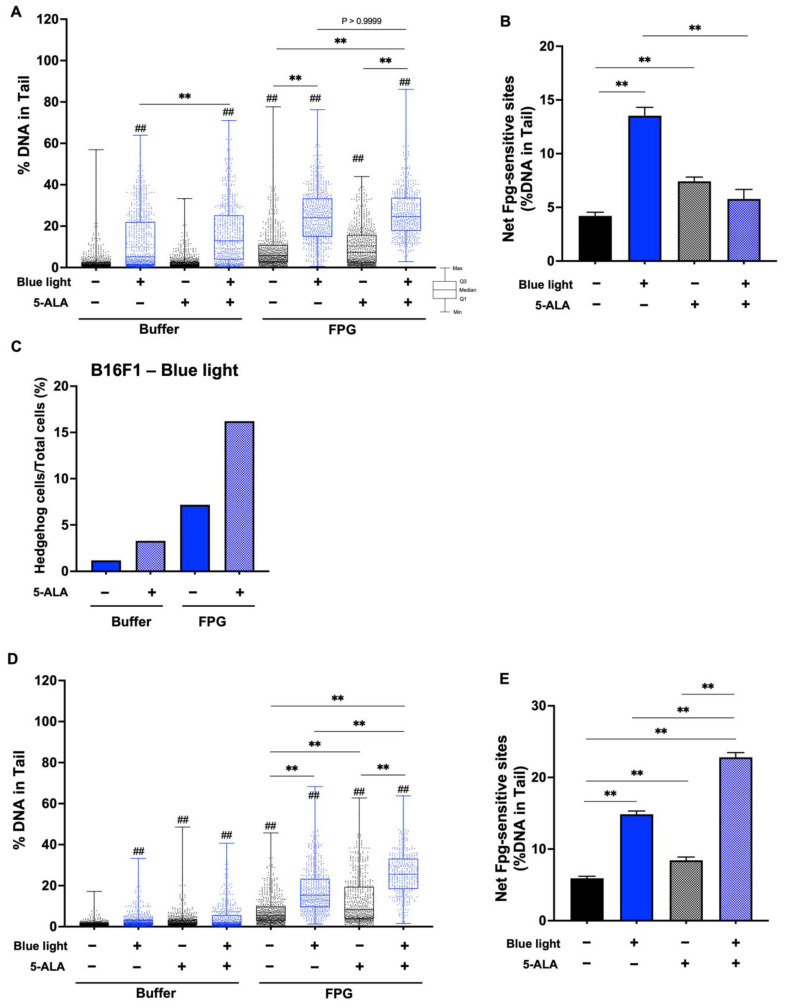
Effect of blue light and 5-ALA on Fpg-sensitive sites in B16F1 and HaCaT cells. The Fpg-modified comet assay was used to detect DSBs, SSBs, ALS, and Fpg-sensitive sites. B16F1 and HaCaT cells were pretreated with 0.2 mM 5-ALA for 1 h followed by irradiation with 50 W/m^2^ blue light for 1 h in the presence of 5-ALA. (**A**) %DNA in the tails were measured by the Fpg-modified comet assay in B16F1 melanoma cells. (**B**) Net Fpg-sites in B16F1 cells are expressed as the subtraction between the data obtained after incubation with Fpg or reaction buffer. (**C**) %Hedgehog cells were calculated from the number of hedgehog cells in the total cells including hedgehog cells. (**D**) %DNA in the tail was measured by the Fpg-modified comet assay in HaCaT cells. (**E**) Net Fpg-sites in HaCaT cells are expressed as the subtraction between the data obtained after incubation with Fpg or reaction buffer. Data are presented as the medians (**A**,**D**) or mean ± SEM (**B**,**E**) obtained from at least four independent experiments. ^##^ *p* < 0.01 versus control, ^##^ *p* < 0.01, ** *p* < 0.01 was considered significant.

**Figure 8:**
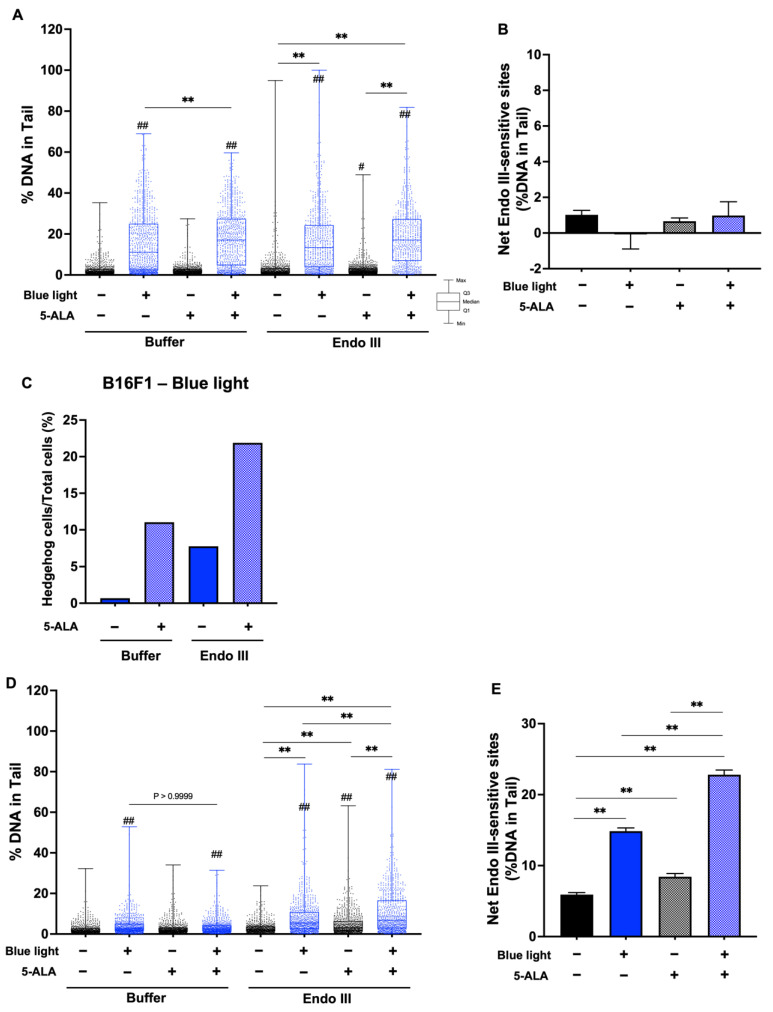
Effect of blue light and 5-ALA on Endo III-sensitive sites in B16F1 and HaCaT cells. The Endo III-modified comet assay was used to detect DSBs, SSBs, ALS, and Endo III-sensitive sites. B16F1 and HaCaT cells were pretreated with 0.2 mM 5-ALA for 1 hfollowed by irradiation with 50 W/m^2^ blue light for 1 h in the presence of 5-ALA. (**A**) %DNA in the tail were measured by the Endo III-modified comet assay in B16F1 melanoma cells. (**B**) Net Endo III sites in B16F1 melanoma cells are expressed as the subtraction between the data obtained after incubation with Endo III or reaction buffer. (**C**) %Hedgehog cells were calculated from the number of hedgehog cells in the total cells including hedgehog cells. (**D**) %DNA in the tail were measured by the Endo III-modified comet assay in HaCaT cells. (**E**) Net Endo III sites in HaCaT cells are expressed as the subtraction between the data obtained after incubation with Endo III or reaction buffer. Data are presented as the medians (**A**,**D**) or mean ± SEM (**B**,**E**) obtained from at least four independent experiments. ^#^ *p* < 0.05, ^##^ *p* < 0.01 versus control, ^##^ *p* < 0.01, ** *p* < 0.01 was considered significant.

To evaluate DNA damage caused by blue light and 5-ALA, we performed an enzyme-modified comet assay. Figure 7A shows the %DNA in the tail data after the Fpg-comet assay in B16F1 melanoma cells, and blue light significantly enhanced %DNA. Fpg-sensitive sites were increased following blue light exposure and 5-ALA-only treated groups were also enhanced significantly compared with the control groups. In the presence of Fpg, there was no difference of %DNA in the tails between blue light-only treated cells and co-treated cells. In contrast, Fpg-sensitive sites were decreased following co-treatment with blue light and 5-ALA compared with the blue light-only irradiated group (Figure 7B). This contradiction may be resolved by considering the markedly higher percentage of hedgehog cells (Figure 7C). In HaCaT cells, Fpg-sensitive sites were enhanced by blue light exposure and blue light-induced damage was increased in the presence of 5-ALA (Figure 7D,E). We also performed the Endo III-modified comet assay. Our data indicated that Endo III-sensitive sites were not significantly altered in B16F1 melanoma cells (Figure 8A,B); however, the percentage of hedgehog cells was increased after blue light and 5-ALA treatment (Figure 8C). Endo III-sensitive sites in HaCaT cells were significantly increased by blue light and 5-ALA treatment (Figure 8D,E).

## 4. Discussion

In this study, we evaluated the effect of blue light on genome integrity in the presence of the photosensitizer 5-ALA. First, we determined its effect on the viability of B16F1 melanoma and HaCaT cells. We showed that exposure to blue light decreased viability in both cell lines. We previously reported that the proliferation rate of B16F1 and B16F10 melanoma cells was significantly inhibited after exposure to photon flux density at 150 µmol m^−2^ s^−1^ of blue light for 15 min [10]. We consider that short-term irradiation of blue light does not cause irreversible cell death, but inhibit cell proliferation arrest. In addition, blue light and 5-ALA co-treatment significantly inhibited cell viability. In contrast, 5-ALA did not exert any cytotoxicity under the experimental condition used in this study. Recently, Ji et al. examined the effect of 5-ALA on the RAW364.7 macrophage cell line and found that 5-ALA exhibited toxicity at high concentrations (>10 mM) [26].

Next, we assessed DSB formation using a neutral comet assay. Our results indicated that after only blue light irradiation for 1 h induced DSB formation, which was markedly increased in the presence of 1 mM 5-ALA in B16F1 melanoma cells. In contrast, DSB formation in HaCaT cells was not enhanced by blue light exposure. Similarly, co-treatment with blue light and 5-ALA markedly increased DSB formation in HaCaT cells. We performed an alkaline comet assay that also reflect SSBs and ALS sites. The results indicated that co-treatment with blue light and 5-ALA significantly induced DNA damage in both cell lines. We also detected highly damaged “hedgehog cells” in co-treated B16F1 melanoma cells. In contrast, hedgehog cells were not observed in HaCaT cells under our experimental conditions. This result implies that HaCaT cells are less sensitive than B16F1 melanoma cells with regard to DNA integrity. However, MTT assay indicated that HaCaT cells were more vulnerable to co-treatment with blue light and 5-ALA. This inconsistency can be likely related to a different sensitivity of cell death pathway. 

We also demonstrated that the TT-comet assay could detect DSB and SSB formation separately in non-sperm cells. Recently, Copp et al., assessed DNA strand breaks in chondrocytes from cadaveric doners using the TT-comet assay [27]. Therefore, this assay may be applicable for the detection of both types of strand breaks in other cell types and tissues. Our method was primarily based on that of Palazzese et al. [20], but with several modifications. We performed the TT-comet assay using B16F1 melanoma cells and HaCaT cells, and the data adequately indicated the level of DNA damage. Although we could clearly observe DSB and SSB damage in the blue light and 5-ALA co-treated cells, numerical differences were obscure compared with the other comet assays. The reason for this discrepancy may rely in our method for data analysis. In the TT-comet assay, we measured the length of the whole comet (including head area), not just the tail length. 

In the enzyme-modified comet assay, the addition of repair enzymes usually causes an increase of %DNA in the tail and other parameters in both cell lines. Using this assay, we did not observe an increase of net Fpg- and Endo III-sensitive sites after blue light and 5-ALA treatment of B16F1 melanoma cells. This may be attributed to the severe damage caused by enzyme treatment in melanoma cells. In fact, the percentage of hedgehog cells was dramatically increased by both Fpg and Endo III treatment in melanoma cells. We also evaluated oxidative DNA damage in HaCaT cells. Both net Fpg- and Endo III-sensitive sites were significantly enhanced following co-treatment. These results indicate that blue light exposure results in oxidized purines and pyrimidines in both cell lines and blue light-induced DNA damage was markedly enhanced in the presence of 5-ALA.

Several reports have provided evidence showing the benefits of 5-ALA treatment or supplementation. 5-ALA can control glucose metabolism and its oral administration alleviates mild hyperglycemia [28,29]. Recently, Yang et al., demonstrated that 5-ALA PDT accelerated the migration of epidermal stem cells and this finding indicates that 5-ALA may be used in clinical applications [30]. Moreover, 5-ALA stimulated murine hair growth in the presence of iron ions [31]. However, our results indicate the adverse effects of blue light were markedly accelerated in the presence of 5-ALA in both cell lines. It is possible that excess ROS accumulation is caused by blue light and 5-ALA in combination, which results in DNA damage and subsequent cell death. It has been shown that heme acts as a free radical generator during PDT treatment [32]. In addition, although we treated cells to avoid light contamination as possible, our data indicated that 5-ALA induced DNA strand breaks and/or oxidative DNA damage. Several reports demonstrated that 5-ALA itself could cause cell damage. Wang et al. showed that 1.5 mM of 5-ALA significantly inhibited cell viability of human fibroblasts [33]. Moreover, Ito et al. assessed the effect of 5-ALA on normal gastric epithelial cells, which revealed that 5-ALA induced caspase 3 activation, p53 phosphorylation, and subsequent apoptosis [6]. 

Although ALA-PDT is an applicable therapy for various cancer cells, solid tumors may fail to produce enough PPIX [34]. As mentioned above, PPIX acts as a photosensitizer during PDT. p53 appears to be involved in the regulation of PPIX concentration [34]. Interestingly, Sznarkowska et al. reported that PPIX can bind to p53/murine double minute 2 (MDM2), stabilize p53, and cause subsequent cell death [35]. Furthermore, they revealed that even p53 null cells underwent apoptosis via disruption of p73/MDM2 complex. Additionally, Anand et al. showed that PPIX level was enhanced by 5-fluorouracil in p53 null cells, and the cells had high sensitivity to PDT [34]. These findings imply some cases of PDT-induced cells death are p53-independent, and provide the methods for efficient anticancer therapy. However, it is important to consider the possibility of normal cell death induction by combination of PPIX-inducer and PDT. 

## 5. Conclusions

Taken together, our data suggest that blue light and 5-ALA co-treatment may cause irreversible cell damage in tumor cells, normal cells, and tissues. This suggests that 5-ALA as a photosensitizer may generate ROS through daily sun exposure. Therefore, the risk of 5-ALA PDT-induced DNA damage should be taken into consideration. In this study, we focused on DNA integrity. Further studies are needed to confirm the effect of 5-ALA on cell metabolism, i.e., other cell death pathways, including apoptosis. 

## Data Availability

All data may be obtained from the authors upon reasonable request.
